# Association between Health-Related Quality of Life and Access to Chronic Disease Management by Primary Care Facilities in Mainland China: A Cross-Sectional Study

**DOI:** 10.3390/ijerph20054288

**Published:** 2023-02-28

**Authors:** Yang Wang, Yibo Wu, Hongling Chu, Zhijie Xu, Xinying Sun, Hai Fang

**Affiliations:** 1 School of Public Health, Peking University, Beijing 100191, China; 2 China Center for Health Development Studies, Peking University, Beijing 100191, China; 3 Research Center of Clinical Epidemiology, Peking University Third Hospital, Beijing 100191, China; 4 Department of General Practice, The Second Affiliated Hospital, Zhejiang University School of Medicine, Hangzhou 310009, China

**Keywords:** primary healthcare, primary care, population health, community health, preventive medicine, chronic disease management, health reform

## Abstract

The integration of chronic disease management (CDM) services into the essential public health services offered by primary care facilities has been a major strategy in China’s healthcare reform since 2009. We aimed to measure the percentage of patients with chronic diseases in China who believed that they could easily obtain CDM services at a nearby primary care facility in mainland China and determine its association with the EQ visual analog scale (EQ-VAS) score and the utility index of the 5-level EQ-5D version (EQ-5D-5L). A cross-sectional survey was conducted nationwide between 20 June 2022 and 31 August 2022, involving 5525 patients with chronic diseases from 32 provincial-level administrative divisions, of which 48.1% (*n* = 2659) were female with a median age of 55.0 years. The median EQ-VAS score was 73.0 and the utility index of the EQ-5D-5L was 0.942. A majority of patients reported definite (24.3%) or mostly (45.9%) easy access to CDM services from nearby primary care facilities. Multivariable logistic regression analysis revealed that easy access to CDM services in primary care facilities was positively associated with higher HRQoL. Our findings indicate that, as of 2022, approximately 70% of patients with chronic diseases in mainland China had easy access to CDM services provided by primary care facilities, which was significantly and positively associated with their health status.

## 1. Introduction

The prevalence of chronic diseases among Chinese residents has experienced a significant increase from 12.8% in 1998 to 34.3% in 2018, and among individuals aged ≥ 65 years the rate has risen from 51.8% to 62.3% [1]. Deaths caused by four major chronic diseases (cardiovascular disease, diabetes, cancer, and chronic respiratory diseases) reached 8.1 million in 2017 [2]. In response, China’s health system reform has implemented, from 2009, a critical strategy for chronic disease management (CDM) in primary care: the addition of CDM services to the essential public health services (EPHS) that are accessible to the majority of residents, primarily provided by primary care facilities, and financed by both central and local governments [3,4].

The primary healthcare system in China consists of two core components: the EPHS and essential medical services. The former is usually managed by public health professionals and nurses, while the latter is provided by primary care clinicians [3]. In some regions only, where the family doctor contract system is relatively well established, the cooperation between the two is relatively close, and the family doctor team formed by various professionals provides integrated primary care services for the contracted residents [3]. This is mainly due to the limited availability of trained general practitioners (GPs), which stood at 2.90 per 10,000 residents until 2020, with 53.9% having no prior experience as GPs in primary care facilities [5,6]. Additionally, primary care facilities in China do not have gatekeeping roles in the healthcare system, and their capacity to provide clinical and preventive care is constrained by historical factors [7].

According to the Essential Public Health Services (EPHS) guidelines, primary care facilities should offer comprehensive diabetes and hypertension management services that comprise regular health education, annual screening, follow-up, treatment, and physical examinations for local residents. Additionally, elderly individuals should receive annual physical examinations and health counseling [8]. In recent years, EPHS guidelines have expanded to include health management for patients with tuberculosis, cancer screening for rural women, and health literacy promotion, which includes reducing smoking rates [9]. Despite the fact that over 90% of China’s population is now covered by the EPHS framework [10], the quality of the services rendered remains inadequate, leading to concerns about the effectiveness of CDM services that lack the full 4Cs features (first contact, comprehensiveness, coordination, and continuity). These features, according to global evidence, serve as the foundation for primary care in achieving improved population health, reduced inequality, and lower costs, and are closely linked to the involvement of trained GPs [11].

Two previous studies conducted in China have investigated the impact of healthcare access on the self-assessed health and quality of life of the elderly population with chronic diseases [12] and revealed a positive association between EPHS coverage and an increased hypertension control rate [13]. However, these studies were conducted prior to, or at the early stages of, China’s health system reform, when the availability of CDM services in the EPHS had not been widely implemented. Besides, health outcomes were measured using a 5-point Likert scale or clinical indicators (such as blood pressure monitoring at a doctor’s office), which have limited capacity to evaluate accurately and comprehensively the health status of a patient as a whole person [12,13,14].

So far, health system reform has been implemented for over a decade, affecting more than 1.4 billion people [15]. Thus, in this study, we further explore the relationship between access to specific CDM services and patient-reported outcomes (PROs), which are patient-centered measures that allow for a more comprehensive assessment of the effects of quality of care on health status from a patient perspective [14]. Specifically, we aim to assess the percentage of patients with chronic diseases in China who perceive that they can easily access CDM services at a nearby primary care facility in 2022. Additionally, the study explores the correlation between the degree of ease of access to CDM services and higher health-related quality of life (HRQoL).

## 2. Materials and Methods

### 2.1. Study Population

Psychology and behavior investigation of Chinese residents (PBICR) was a nationwide survey covering 32 provincial administrative units in mainland China, conducted between 20 June 2022 and 31 August 2022. A total of 21,916 residents living in 780 communities/villages were included in the PBICR survey using stratified sampling and quota sampling methods. The survey collected personal/family information, personal health status, social environment, psychological level, and behavioral level data from all participants [16]. In the personal health status section, it investigated whether participants had the following chronic conditions: stroke, coronary heart disease, dyslipidemia, cancer, chronic respiratory disease, diabetes, chronic kidney disease, chronic gastritis/enteritis, viral hepatitis, fatty liver disease, Alzheimer’s disease, Parkinson’s disease, mood disorders (anxiety, depression, etc.), cataract, osteoporosis, arthritis, and others.

The survey was conducted through a public-health academic network that encompasses multiple provincial administrative units in China. Within each unit, researchers from the public health departments of universities or related institutes established a survey network consisting of multiple survey sites by cooperating with local community health centers or sub-district offices situated in the communities. During the survey period, trained investigators recruited a specified number of local residents who met the inclusion criteria in each site according to a quota. This recruitment was achieved through the use of posters and face-to-face conversations, and the recruited individuals were then guided to fill out the electronic questionnaire by mobile phone or tablet. The complete questionnaires were automatically documented in a back-end server. According to the final statistics, the response rate of the questionnaire was 71.8%. More details about the study design have been previously reported [16].

The inclusion criteria for PBICR survey participants who were enrolled into the present study were: (a). living permanently in mainland China (travel outside of China ≤1 month/year); (b). who self-reported suffering from at least one chronic disease based on clinical diagnosis by clinicians; (c). age ≥ 18 years. The data were analyzed from 5 September 2022 to 12 October 2022.

### 2.2. Access to CDM Services Provided by Primary Care Facilities

The degree of easy access to CDM services provided by primary care facilities was the independent variable. It was measured using a pre-determined question, namely, “How would you assess: nearby primary care facility makes it easy for me to get CDM service”, and respondents could choose to answer “definitely”, “mostly”, “somewhat”, or “not at all” (from highest to lowest). This question was adapted from the first question of the Chinese version of the Person-Centered Primary Care Measure (PCPCM), which is used to measure the accessibility of primary care [17]. The PCPCM is an 11-item patient-reported tool that assesses vital functions of primary care and the psychometric properties of 35 countries [18]. The Chinese version of the PCPCM was translated and tested for cultural adaptability, content validity, and psychometric properties in Hong Kong by Tse et al. [17,19].

However, due to the significant differences between primary care in mainland China and in Hong Kong (e.g., most patients in mainland China do not have a stable primary care physician, but are free to choose and access various specialists in different hospitals [7]), after consulting with a group of primary care and public health experts in China, and considering the purpose of our study and the characteristics of the CDM services in the EPHS, we dropped the use of the full PCPCM to assess other aspects of primary care in mainland China, such as comprehensiveness, integration, coordination, and relationship, and added only the modified first question of the PCPCM in this survey.

### 2.3. Health-Related Quality of Life

The outcome variables of this study were the EQ visual analog scale (EQ-VAS) score and the utility index of the 5-level EQ-5D version (EQ-5D-5L). The EQ-5D-5L is a widely used multi-attribute utility instrument (MAUI) with Patient-Reported Outcome Measures (PROMs) that assess respondents’ health-related quality of life. It encompasses five dimensions (mobility, self-care, usual activities, pain/discomfort, anxiety/depression), with each dimension having five response levels (no problems, slight, moderate, severe, and extreme problems) [14,20]. The EQ-VAS is a visual analog scale included in the EQ-5D, which provides a self-perceived health status on a scale of 0 to 100, with 100 indicating the best HRQoL [21]. We used population weighs for China to convert the EQ-5D-5L response into the EQ-5D utility index [22]. This index considers each level reported on each dimension as a unique health-state weight, values full health as 1 and death as 0, and reflects people’s preferences about how good or bad their health state is [21].

### 2.4. Covariates

Based on determinants of health and previous findings [11,23], we used the following covariates, which were theoretically associated with the health status of participants: sex (male/female), age (years), region (eastern region, central region, western region, and northeast region), residential area (rural/urban), education (elementary school or lower, middle school, high school, college/bachelor’s degree or higher), working status (working or studying, retirement processed, unemployed or without a regular job), injury in the last year (yes/no), subjective socioeconomic status (Level 1 to 7, with Level 1 being the lowest), monthly income (0–2000, 2001–4000, 4001–6000, 6000 or higher; unit: yuan), medical insurance (yes/no), alcohol consumption (yes/no), smoking (never smoke, quitted smoking, smoking), can get social support from family (from “very strongly disagree” to “very strongly agree”, 7 levels), and body mass index (BMI). The subjective socioeconomic status was measured using a seven-point Likert scale in Chinese adapted from the MacArthur Scale of Subjective Social Status, which was tested in the local population [24,25].

### 2.5. Statistical Analysis

Descriptive statistics were used to report the prevalence of chronic diseases among residents, sociodemographic characteristics, the degree of easy access to CDM services provided by primary care facilities, and the HRQoL of all the patients. For continuous variables, we report means and 95% confidence interval (CI) or median and interquartile range (IQR), while percentages are reported for categorical variables. Student’s *t*-test and non-parametric tests or chi-square tests were used to compare the differences in sociodemographic characteristics and HRQoL between two groups of patients: easy-access (individuals who chose “definitely” or “mostly”) and not-easy-access (individuals who chose “somewhat” or “not at all”) with respect to CDM services provided by primary care facilities.

The association between the degree of easy access to CDM services and the EQ-VAS score/utility index of the EQ-5D-5L was examined by a univariate linear regression model and a multivariate linear regression model. We also examined the association between all covariates with *p* ≤ 0.2 in sub-group comparison and outcome variables in several univariate linear regression models. All the covariates with *p* ≤ 0.2 in the univariate regression and without multicollinearity were included in the multivariate linear regression model. We applied the stepwise method using a backward hierarchical approach to select variables with *p*-value < 0.2 from one level to another. We used robust standard errors to control for heteroskedasticity and the White test to test the homoskedasticity assumption. We also used the variance inflation factor (VIF) to measure multicollinearity and excluded variables with VIF ≥ 10. Statistical analysis was conducted using Stata software, version 17.0 SE (StataCorp), and the significance level was set at *p* ≤ 0.05.

### 2.6. Ethical Considerations

The PBICR survey was conducted in accordance with the Declaration of Helsinki. Written consent was obtained from all the patients prior to commencement of the study. It was approved by the Ethics Research Committee of the 2nd Xiangya Hospital of Central South University (No. 2022-K050).

## 3. Results

### 3.1. Sociodemographic Characteristics and HRQoL

A total of 5525 patients with chronic diseases were eligible for this study from a total of 21,916 residents. Among them, 3565 (64.5%) had a single chronic disease, while 1960 (35.5%) had multimorbidity. Table 1 shows the sociodemographic characteristics and the HRQoL of patients. Female patients accounted for 48.1% (2659) of the study population. Their median age was 55.0 years (IQR, 42–67 years), and the mean BMI was 22.1 (95% CI: 22.0–22.3).

**Figure 1:**
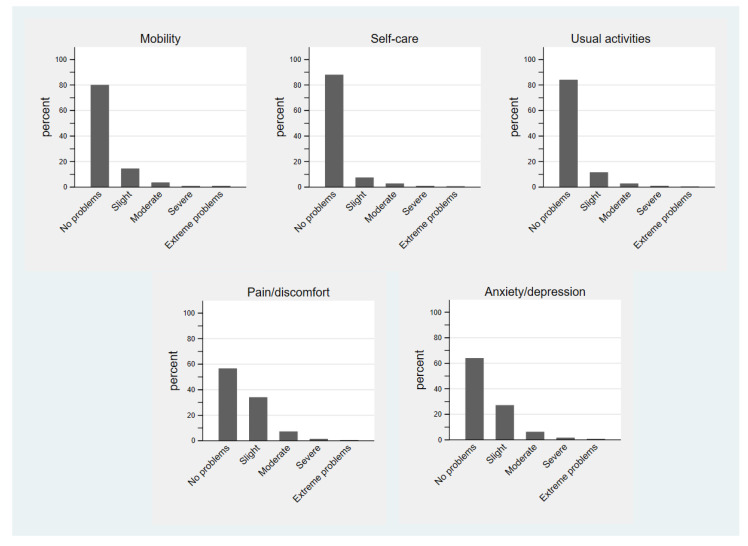
Dimensions of EQ-5D-5L for patients with chronic diseases. EQ-5D-5L, 5-level EQ-5D version.

The median EQ-VAS score and the utility index of the EQ-5D-5L were 73.0 (IQR, 59.0, 84.0) and 0.942 (IQR, 0.876, 1.000), respectively. Regarding the EQ-5D-5L, the majority of patients reported no problems in mobility (80.0%), self-care (88.0%), usual activities (84.1%), pain/discomfort (56.6%), or anxiety/depression (64.1%) (Figure 1).

### 3.2. Comparison of HRQoL between Easy-Access and Not-Easy-Access Groups

**Figure 2:**
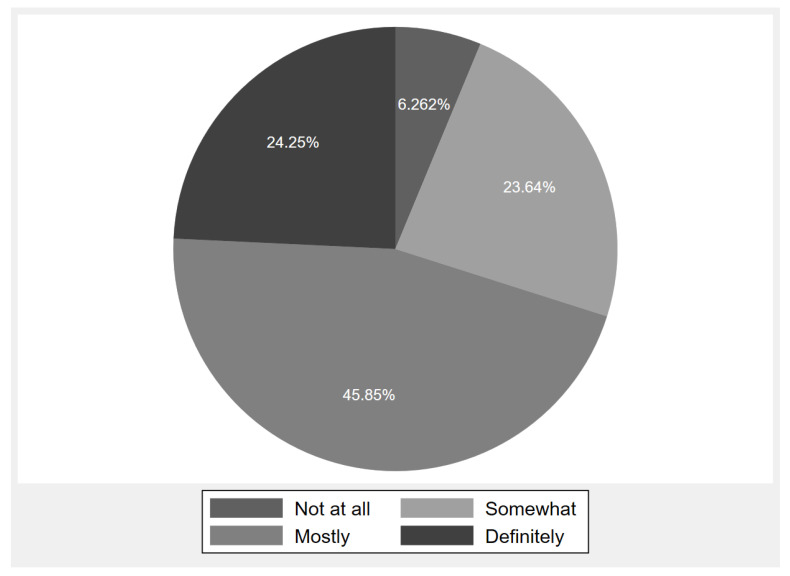
Percentage of patients who can easily obtain chronic disease management services at a nearby primary care facility.

The majority of patients reported that they had definitely (24.3%) and mostly (45.9%) easy access to CDM services from nearby primary care facilities (Figure 2). Table 1 shows the differences in sociodemographic characteristics and HRQoL between easy-access and not-easy-access groups. Compared to the not-easy-access group, the easy-access group showed a significantly higher percentage for the following variables: males (52.9% vs. 49.6%, *p* = 0.028), living in the eastern region (34.3% vs. 27.7%, *p* ≤ 0.001), urban area (67.0% vs. 63.4%, *p* = 0.01), college/Bachelor’s degree or higher education level (38.3% vs. 34.4%, *p* = 0.008), working, studying, or retirement processed (69.7% vs. 63.4%, *p* ≤ 0.001), no injury in the past year (81.6% vs. 78.0%, *p* ≤ 0.001), Level 5–7 subjective socioeconomic status (44.6% vs. 34.7%, *p* ≤ 0.001), monthly income higher than 4000 yuan (49% vs. 37.8%, *p* ≤ 0.001), have medical insurance (96.8% vs. 94.4, *p* ≤ 0.001), smoking (22.0% vs. 18.4%, *p* = 0.009), and strongly or very strongly agree that can get social support from family (47.3% vs. 39.5%, *p* ≤ 0.001).

The HRQoL of the easy-access group was significantly higher than that of the not-easy-access group (EQ-VAS score: 75.0 [60.0–85.0] vs. 70.0 [53.0–81.0], *p* ≤ 0.001; utility index of EQ-5D-5L: 0.951 [0.893–1.000] vs. 0.942 [0.827–1.000], *p* ≤ 0.001).

### 3.3. Association between Easy Access to CDM Services and HRQoL

In the univariate linear regression models, the degree of easy access to CDM services provided by primary care facilities was significantly and positively associated with a higher EQ-VAS score and a higher utility index of EQ-5D-5L (Table 2).

We included in the multivariate linear regression model only covariates with *p* ≤ 0.2 in the univariate linear regression model. Moreover, no collinearity was observed in all the independent variables and covariates. Therefore, we excluded age in the two models. In the model with the utility index of the EQ-5D-5L as the outcome variable, we also excluded drinking. With the adjustment of covariates, this positive association remained in the two multiple linear regression models. The R^2^ of the multivariate linear regression model was 0.14 and 0.12 (Table 3).

## 4. Discussion

In this study, we evaluated an important primary care and CDM strategy in China’s healthcare reform since 2009 from the patient’s perspective. Our results revealed that in 2022, the majority of patients (70.2%) with chronic diseases in mainland China reported that they had “definitely” and “mostly” easy access to CDM services in nearby primary care facilities. Moreover, the degree of ease of access to this service was positively associated with higher HRQoL.

The extant literature offers evidence of the relationship between accessibility to healthcare and health status [11,26,27]. This study has further elucidated the association between the degree of easy access to CDM services in nearby primary care facilities with PRO (HRQoL). The implementing institution, context, and content of these services encompass significant preventive medicine features, especially secondary and tertiary prevention at the community level, which can help achieve early management of health problems and create cumulative effects on health over time. This mechanism has been confirmed by evidence from primary care studies in multiple countries [28]. Our study demonstrates that prevention-oriented CDM services implemented in primary care, even without full 4Cs features and with limited involvement of experienced GPs, may be associated with improvements in population health and may alleviate health inequalities due to differences in social factors, such as social status, education, income, living region, and family support.

In practice, health expenditure in low- and middle-income countries (LMICs) is significantly lower than that in high-income countries, which limits the scope of primary care services and the qualification/capacity of primary care practitioners in LMICs [29]. Our study demonstrates that better access to CDM services provided by public health practitioners and nurses in primary care facilities in China might also be associated with better health outcomes. Two other studies conducted in South Africa [30] and Ethiopia [31] also demonstrated that CDM services provided by nurses in the primary care facilities of LMICs are feasible and cost-effective. These findings demonstrate that in LMICs with limited resources, enhancing targeted long-term national investments in specific health services with universal coverage of residents and implemented by public primary care facilities may improve population health. The mechanism for delivery of such an outcome may be a practical stepwise improvement of primary care. The focus should be firstly on achieving a few specific features of good primary care (e.g., accessibility) to help address key health risk factors, followed by gradual refinement and development of the system to respond effectively to the population needs.

However, this approach has also shown limitations at this stage. A previous study reported that, despite the Chinese government’s tenfold increase in annual investment in primary healthcare and the fact that the essential public health services (EPHS) framework covers approximately 90% of Chinese residents, the effect on health education and clinical outcomes for patients with hypertension and diabetes was limited [32]. This finding highlights the importance of expanding clinical sections and involving qualified primary care practitioners in further primary healthcare reform. Additionally, more effort is needed to alleviate health inequalities; although the EPHS framework covers the majority of residents, our study revealed that some patients still could not easily obtain Chronic Disease Management (CDM) services from nearby primary care facilities. This may demonstrate inequity in the distribution of primary care facilities across different regions and in the supply of CDM services to vulnerable populations. For example, even in Beijing, which has the highest density of medical resources in China [33], the spatial accessibility of primary healthcare services for 22.8% of the community remains low [34]. In mainland China, the main population that chooses healthcare services in primary care facilities is residents of less economically developed regions with low income and education levels [35]; however, our study found that this population had a lower degree of easy access to CDM services provided by primary care facilities compared to the average population.

Another key strategy in recent healthcare reform in China is the training of large numbers of GPs and traditional Chinese medicine doctors to work in primary care facilities and the encouragement of patients to voluntarily sign a family doctor contract with a primary care clinician. This approach theoretically allows patients to receive comprehensive, continuous, and coordinated health management services [36]. In future research, we will assess the impact of this new service in conjunction with the CDM services available in the EPHS on the patients’ health status.

This study has three main limitations. First, the self-reported ease of access to CDM services is closely related to, but not necessarily equivalent to, the CDM component of the EPHS. To make it easier for respondents to understand the question and provide realistic answers, we did not mention the EPHS directly in the questions, but instead referred to obtaining CDM services in nearby primary care facilities. Second, the cross-sectional survey only demonstrated an association between exposure and outcome, but not a clear causal relationship between the degree of easy access and improvement in health status. Third, survey data may have limited accuracy and objectivity, but a large sample survey is a realistic and practical approach in the absence of a national primary care research network in mainland China.

## 5. Conclusions

In this nationally representative study conducted in mainland China, we observed that in 2022, approximately 70% of patients with chronic disease reported that they had “definitely” and “mostly” easy access to CDM services in nearby primary care facilities. Moreover, the degree of easy access to these services was positively correlated with better health status.

## Figures and Tables

**Table 1:** Patients’ sociodemographic characteristics for the easy-access and not-easy-access groups with respect to CDM services provided by primary care facilities.

Characteristics	All (*n* = 5525)	Not Easy-Access Group	Easy-Access Group	*p*-Value
**Gender, No (%)**				0.028
Female	2659 (48.1)	833 (50.4)	1826 (47.1)	
Male	2866 (51.9)	819 (49.6)	2047 (52.9)	
**Age, median (IQR), y**	55.0 (42–67)	55.0 (42.0–67.0)	55.0 (43.0–67.0)	0.360
**BMI, mean (95% CI)**	22.1 (22.0–22.3)	22.1 (21.8–22.3)	22.2 (22.0–22.3)	0.510
**Region, No (%)**				≤0.001
Eastern region	1772 (32.1)	457 (27.7)	1315 (34.2)	
Central region	1079 (19.5)	331 (20.1)	748 (19.4)	
Western region	2383 (43.1)	766 (46.4)	1617 (42.0)	
Northeast region	266 (4.8)	95 (5.8)	171 (4.4)	
**Living area, No (%)**				0.010
Urban	3644 (66.0)	1048 (63.4)	2596 (67.0)	
Rural	1881 (34.0)	604 (36.6)	1277 (33.0)	
**Education, No (%)**				0.008
Elementary school or lower	1549 (28.0)	510 (30.9)	1039 (26.8)	
Middle school	1230 (22.3)	362 (21.9)	868 (22.4)	
High school	695 (12.6)	212 (12.8)	483 (12.5)	
College/Bachelor’s degree or higher	2051 (37.12)	568 (34.4)	1483 (38.3)	
**Work, No (%)**				≤0.001
Working or studying	2202 (39.9)	645 (39.0)	1557 (40.2)	
Retirement processed	1546 (28.0)	403 (24.4)	1143 (29.5)	
Unemployed or without a regular job	1777 (32.2)	604 (36.6)	1173 (30.3)	
**Suffered injury, No (%)**				≤0.001
Yes	1078 (19.5)	364 (22.0)	714 (18.4)	
No	4447 (80.5)	1288 (78.0)	3159 (81.6)	
**Subjective socioeconomic status, No (%)**				≤0.001
Level 1	95 (1.7)	41 (2.5)	54 (1.4)	
Level 2	310 (5.6)	137 (8.3)	173 (4.5)	
Level 3	914 (16.5)	320 (19.4)	594 (15.3)	
Level 4	1905 (34.5)	579 (35.1)	1326 (34.2)	
Level 5	1419 (25.7)	367 (22.2)	1052 (27.2)	
Level 6	596 (10.8%)	146 (8.8)	450 (11.6)	
Level 7	286 (5.2)	62 (3.7)	224 (5.8)	
**Monthly income, No (%)**				
0–2000 yuan	1183 (21.4)	446 (27.0)	737 (19.0)	≤0.001
2001–4000 yuan	1820 (32.9)	581 (35.2)	1239 (32.0)	
4001–6000 yuan	1296 (23.5)	314 (19.0)	982 (25.4)	
6000 yuan or higher	1226(22.2)	311 (18.8)	915 (23.6)	
**Have medical insurance, No (%)**				≤0.001
Yes	5310 (96.1)	1560 (94.4)	3750 (96.8)	
No	215 (3.9)	92 (5.6)	123 (3.2)	
**Drinking, No (%)**				0.105
Yes	1297 (22.8)	353 (21.4)	905 (23.4)	
No	4267 (77.2)	1299 (78.6)	2968 (76.6)	
**Smoking, No (%)**				0.009
Never smoking	3968 (71.8)	1217 (73.7)	2751 (71.0)	
Quitted smoking	403 (7.3)	131 (7.9)	272 (7.0)	
Smoking	1154 (20.9)	304 (18.4)	850 (22.0)	
**Social support—family, No (%)**				≤0.001
Very strongly disagree	103 (1.9)	24 (1.5)	79 (2.0)	
Strongly disagree	148 (2.7)	65 (3.9)	83 (2.1)	
Mildly disagree	410 (7.4)	134 (8.1)	276 (7.1)	
Neutral	1196 (21.7)	399 (24.1)	797 (20.6)	
Mildly agree	1188 (21.5)	378 (22.9)	810 (20.9)	
Strongly agree	1754 (31.8)	474 (28.7)	1280 (33.1)	
Very strongly agree	726 (13.1)	178 (10.8)	548 (14.2)	
**EQ-VAS score, median (IQR)**	73.0 (59.0–84.0)	70.0 (53.0–81.0)	75 (60.0–85.0)	≤0.001
**Utility index of EQ-5D-5L, median (IQR)**	0.942 (0.876–1.000)	0.942 (0.827–1.000)	0.951 (0.893–1.000)	≤0.001

BMI, body mass index; CI, confidence interval; EQ-5D-5L, 5-level EQ-5D version; EQ-VAS, EQ visual analog scale; IQR, interquartile range.

**Table 2:** Univariate linear regression: by EQ-VAS score and utility index of EQ-5D-5L.

	Coefficient [95% CI]	
	EQ-VAS Score	Utility Index of EQ-5D-5L
**Degree of easy access to the CDM service**		
Not at all	Reference	Reference
Somewhat	2.96 ** [0.62, 5.31]	0.0388 ** [0.0187, 0.0589]
Mostly	5.71 ** [3.49, 7.93]	0.0634 ** [0.0443, 0.0825]
Definitely	8.10 ** [5.76, 10.43]	0.0844 ** [0.0644, 0.1045]
**Gender**		
Female	Reference	Reference
Male	0.02 [−1.03, 1.06]	−0.0031 [−0.0122, 0.0059]
**Region**		
Eastern region	Reference	Reference
Central region	1.37 * [−0.13, 2.87]	0.0019 [−0.0110, 0.0148]
Western region	−1.87 ** [−3.08, −0.65]	0.0036 [−0.0069, 0.0141]
Northeast region	0.63 [−1.93, 3.19]	−0.0118 [−0.0338, 0.0101]
**Living area**		
Urban	Reference	Reference
Rural	−1.58 ** [−2.69, −0.48]	−0.0086 * [−0.0181, 0.0009]
**Education**		
Elementary school or lower	Reference	Reference
Middle school	3.51 ** [2.04, 4.99]	0.0321 ** [0.0193, 0.0448]
High school	5.37 ** [3.61, 7.13]	0.0296 ** [0.0144, 0.0449]
College/Bachelor’s degree or higher	5.86 ** [4.56, 7.16]	0.0360 ** [0.0248, 0.0472]
**Work**		
Working or studying	Reference	Reference
Retirement processed	−2.47 ** [−3.76, −1.19]	−0.0454 ** [−0.0565, −0.0344]
Unemployed or without a regular job	−3.63 ** [−4.87, −2.40]	−0.0269 ** [−0.0375, −0.0163]
**Suffered injury**		
No	Reference	Reference
Yes	−3.41 ** [−4.73, −2.10]	−0.0880 ** [−0.0991, −0.0768]
**Subjective socioeconomic status of family**		
Level 1	Reference	Reference
Level 2	3.96 * [−0.55, 8.46]	0.0350 * [−0.0042, 0.0742]
Level 3	5.56 ** [1.42, 9.70]	0.0404 ** [0.0044, 0.0764]
Level 4	7.77 ** [3.73, 11.80]	0.0606 ** [0.0255, 0.0957]
Level 5	10.26 ** [6.19, 14.33]	0.0706 ** [0.0352, 0.1060]
Level 6	12.44 ** [8.20, 16.68]	0.0451 ** [0.0082, 0.0820]
Level 7	14.22 ** [9.66, 18.77]	0.0644 ** [0.0248, 0.1039]
**Monthly income**		
0–2000 yuan	Reference	Reference
2001–4000 yuan	2.85 ** [1.41, 4.30]	0.0263 ** [0.0138, 0.0388]
4001–6000 yuan	4.25 ** [2.69, 5.81]	0.0379 ** [0.0245, 0.0514]
6000 yuan or higher	5.10 ** [3.52, 6.68]	0.0270 ** [0.0134, 0.0406]
**Have medical insurance**		
No	Reference	Reference
Yes	4.00 ** [1.29, 6.70]	0.0231 * [−0.0002, 0.0464]
**Drinking**		
No	Reference	Reference
Yes	0.97 * [−0.28, 2.22]	−0.0053 [−0.0160, 0.0055]
**Smoking**		
Never smoked	Reference	Reference
Quitted smoking	−1.18 [−3.22, 0.85]	−0.0777 ** [−0.0951, −0.0604]
Smoking	−3.64 ** [−4.94, −2.34]	−0.0489 ** [−0.0600, −0.0378]
**Social support—family**		
Very strongly disagree	Reference	Reference
Strongly disagree	−3.57 * [−8.30, 1.16]	0.0054 [−0.0369, 0.0478]
Mildly disagree	0.78 [−3.28, 4.85]	−0.0032 [−0.0396, 0.0332]
Neutral	5.20 ** [1.41, 8.98]	0.0408 ** [0.0069, 0.0747]
Mildly agree	9.27 ** [5.49, 13.06]	0.0495 ** [0.0156, 0.0834]
Strongly agree	15.79 ** [12.06, 19.53]	0.0899 ** [0.0564, 0.1234]
Very strongly agree	18.56 ** [14.68, 22.44]	0.0856 ** [0.0509, 0.1204]

CDM, chronic disease management; CI, confidence interval; EQ-5D-5L, 5-level EQ-5D version; EQ-VAS, EQ visual analog scale. * *p* < 0.20, ** *p* < 0.05

**Table 3:** Multivariate linear regression: by EQ-VAS score and utility index of EQ-5D-5L.

	Coefficient [95% CI]	
	EQ-VAS Score	Utility Index of EQ-5D-5L
**Degree of easy access to the CDM service**		
Not at all	Reference	Reference
Somewhat	2.15 [−0.38, 4.68]	0.0310 * [0.0049, 0.0572]
Mostly	3.93 ** [1.51, 6.36]	0.0513 *** [0.0260, 0.0766]
Definitely	4.76 *** [2.17, 7.34]	0.0673 *** [0.0414, 0.0932]
**Region**		
Eastern region	Reference	Reference
Central region	1.79 * [0.33, 3.25]	−0.0026 [−0.0149, 0.0096]
Western region	−1.31 * [−2.49, −0.13]	0.0023 [−0.0078, 0.0124]
Northeast region	1.62 [−1.02, 4.26]	−0.0040 [−0.0271, 0.0191]
**Living area**		
Urban	Reference	Reference
Rural	0.03 [−1.01, 1.07]	0.0003 [−0.0090, 0.0097]
**Education**		
Elementary school or lower	Reference	Reference
Middle school	1.92 ** [0.53, 3.31]	0.0238 *** [0.0109, 0.0367]
High school	3.68 *** [1.98, 5.39]	0.0160 * [0.0002, 0.0318]
College/Bachelor’s degree or higher	3.33 *** [1.71, 4.95]	0.0129 [−0.0018, 0.0275]
**Work**		
Working or studying	Reference	Reference
Retirement processed	−1.67 * [−3.03, −0.32]	−0.0483 *** [−0.0607, −0.0359]
Unemployed or without a regular job	−0.31 [−1.85, 1.22]	−0.0199 ** [−0.0326, −0.0073]
**Suffered injury**		
No	Reference	Reference
Yes	−1.90 ** [−3.20, −0.60]	−0.0761 *** [−0.0901, −0.0621]
**Subjective socioeconomic status**		
Level 1	Reference	Reference
Level 2	3.83 [−2.33, 9.99]	0.0329 [−0.0162, 0.0820]
Level 3	4.46 [−1.31, 10.23]	0.0315 [−0.0153, 0.0783]
Level 4	5.92 * [0.22, 11.62]	0.0460 * [0.0002, 0.0919]
Level 5	6.97 * [1.25, 12.70]	0.0497 * [0.0037, 0.0956]
Level 6	9.54 ** [3.70, 15.37]	0.0270 [−0.0208, 0.0748]
Level 7	10.94 *** [4.74, 17.13]	0.0416 [−0.0084, 0.0916]
**Monthly income**		
0–2000 yuan	Reference	Reference
2001–4000 yuan	1.44 [−0.01, 2.89]	0.0183 ** [0.0049, 0.0318]
4001–6000 yuan	1.68 * [0.04, 3.32]	0.0250 *** [0.0107, 0.0393]
6000 yuan or higher	1.61 [−0.16, 3.39]	0.00721 [−0.0086, 0.0230]
**Have medical insurance**		
No	Reference	Reference
Yes	1.01 [−1.88, 3.92]	−0.0003 [−0.0247, 0.0240]
**Drinking**		
No	Reference	
Yes	1.63 * [0.34, 2.91]	
**Smoking**		
Never smoked	Reference	Reference
Quitted smoking	−1.36 [−3.34, 0.62]	−0.0700 *** [−0.0917, −0.0483]
Smoking	−2.75 *** [−4.11, −1.39]	−0.0369 *** [−0.0487, −0.0252]
**Social support—family**		
Very strongly disagree	Reference	Reference
Strongly disagree	−2.85 [−9.94, 4.24]	0.0009 [−0.0578, 0.0595]
Mildly disagree	1.93 [−4.29, 8.15]	−0.0032 [−0.0545, 0.0481]
Neutral	6.02 * [0.01, 12.02]	0.0309 [−0.0173, 0.0790]
Mildly agree	9.92 ** [3.94, 15.91]	0.0380 [−0.0104, 0.0865]
Strongly agree	15.72 *** [9.77, 21.68]	0.0694 ** [0.0219, 0.1170]
Very strongly agree	17.81 *** [11.72, 23.90]	0.0632 * [0.0149, 0.1114]
**Observation**	5471	5500
**R^2^**	0.14	0.12

CDM, chronic disease management; CI, confidence interval; EQ-5D-5L, 5-level EQ-5D version; EQ-VAS, EQ visual analog scale. * *p* < 0.05, ** *p* < 0.01, *** *p* < 0.001.

## Data Availability

The data presented in this study are available on request from the corresponding author.

## References

[1] National Health Commission of the PRC China Health Statistics Yearbook. http://www.nhc.gov.cn/mohwsbwstjxxzx/tjtjnj/new_list.shtml.

[2] Yin P., Qi J., Liu Y., Liu J., You J., Wang L., Zhou M. (2019). Incidence, Prevalence, and Mortality of Four Major Chronic Non-communicable Diseases—China, 1990–2017. China CDC Wkly..

[3] Meng Q., Mills A., Wang L., Han Q. (2019). China’s Health System Reforms: Review of 10 years of progress: What can we learn from China’s health system reform?. BMJ.

[4] Yuan B., Balabanova D., Gao J., Tang S., Guo Y. (2019). Strengthening public health services to achieve universal health coverage in China. BMJ.

[5] National Health Commission of the PRC Statistical Bulletin on the Development of Health Care in China in 2020. http://www.gov.cn/guoqing/2021-07/22/content_5626526.htm2021.

[6] Wu B.L., Gong H.X., Luo Z.N. (2018). Number, distribution and predicted needed number of general practitioners in China. Chin. Gen. Pract..

[7] Wu D., Lam T.P. (2016). Underuse of Primary Care in China: The Scale, Causes, and Solutions. J. Am. Board Fam. Med..

[8] National Health Commission of the PRC National Basic Public Health Service Guideline 2017 (Third Edition). http://www.nhc.gov.cn/ewebeditor/uploadfile/2017/04/20170417104506514.pdf.

[9] The State Council Information Office of the People’s Republic of China Development of China’s Public Health an Essential Element of Human Rights 2017. http://en.nhc.gov.cn/2019-04/29/c_75161.htm.

[10] Tian M., Wang H., Tong X., Zhu K., Zhang X., Chen X. (2015). Essential Public Health Services’ Accessibility and its Determinants among Adults with Chronic Diseases in China. PLoS ONE.

[11] Zhang T., Liu C., Ni Z. (2019). Association of Access to Healthcare with Self-Assessed Health and Quality of Life among Old Adults with Chronic Disease in China: Urban Versus Rural Populations. Int. J. Environ. Res. Public Health.

[12] Jimenez G., Matchar D., Koh G.C.H., Tyagi S., van der Kleij R.M.J.J., Chavannes N.H., Car J. (2021). Revisiting the four core functions (4Cs) of primary care: Operational definitions and complexities. Prim. Health Care Res. Dev..

[13] Zhang D., Pan X., Li S., Liang D., Hou Z., Li Y., Shi L. (2017). Impact of the National Essential Public Health Services Policy on Hypertension Control in China. Am. J. Hypertens..

[14] Weldring T., Smith S.M. (2013). Article Commentary: Patient-Reported Outcomes (PROs) and Patient-Reported Outcome Measures (PROMs). Health Serv. Insights.

[15] Population, Total-China Data. https://data.worldbank.org/indicator/SP.POP.TOTL?locations=CN.

[16] Wang Y.J., Kaierdebieke A., Fan S.Y., Zhang R.F., Huang M.J., Li H., Sun X., Li Q., Meng W., Wu W. (2022). Study protocol: A cross-sectional study on psychology and behavior investigation of Chinese residents, PBICR. Psychosom. Med. Res..

[17] Tse E.T.Y., Lam C.L.K., Wong C.K.H., Chin W.Y., Etz R.S., Zyzanski S.J., Stange K.C. (2020). Cultural adaptation and content validity of a Chinese translation of the ‘Person-Centered Primary Care Measure’: Findings from cognitive debriefing. Fam. Med. Community Health.

[18] Zyzanski S.J., Gonzalez M.M., O’Neal J.P., Etz R.S., Reves S.R., Stange K.C. (2021). Measuring Primary Care Across 35 OECD Countries. Ann. Fam. Med..

[19] Tse E.T.Y., Lam C.L.K., Wong C.K.H., Chin W.Y., Etz R.S., Zyzanski S.J., Stange K.C. (2021). Exploration of the psychometric properties of the Person-Centred Primary Care Measure (PCPCM) in a Chinese primary care population in Hong Kong: A cross-sectional validation study. BMJ Open.

[20] Herdman M., Gudex C., Lloyd A., Janssen M., Kind P., Parkin D., Bonsel G., Badia X. (2011). Development and preliminary testing of the new five-level version of EQ-5D (EQ-5D-5L). Qual. Life Res..

[21] EuroQol Office EQ-5D Terminology. https://euroqol.org/support/terminology/.

[22] Luo N., Liu G., Li M., Guan H., Jin X., Rand-Hendriksen K. (2017). Estimating an EQ-5D-5L Value Set for China. Value Health.

[23] World Health Organization Determinants of Health. https://www.who.int/news-room/questions-and-answers/item/determinants-of-health.

[24] Xu L., Lin C., Liu L., Yang M.G. (2012). The Influence of Family Social Status on Junior Students’ Choice Motivation in the Context of Financial Crisis: The Mediating Role of Social Support Matching Tendency. Psychology.

[25] Adler N.E., Epel E.S., Castellazzo G., Ickovics J.R. (2000). Relationship of subjective and objective social status with psychological and physiological functioning: Preliminary data in healthy, White women. Health Psychol..

[26] Alonso F.O.A.J., Orfila F., Ruigómez A., Ferrer M., Antó J.M. (1997). Unmet health care needs and mortality among Spanish elderly. Am. J. Public Health.

[27] Porell F.W., Miltiades H.B. (2001). Access to care and functional status change among aged Medicare beneficiaries. J. Gerontol. Ser. B Psychol. Sci. Soc. Sci..

[28] Starfield B., Shi L., Macinko J. (2005). Contribution of Primary Care to Health Systems and Health. Milbank Q..

[29] Kruk M.E., Porignon D., Rockers P.C., Van Lerberghe W. (2010). The contribution of primary care to health and health systems in low- and middle-income countries: A critical review of major primary care initiatives. Soc. Sci. Med..

[30] Coleman R., Gill G., Wilkinson D. (1998). Noncommunicable disease management in resource-poor settings: A primary care model from rural South Africa. Bull. World Health Organ..

[31] Mamo Y., Seid E., Adams S., Gardiner A., Parry E. (2007). A primary healthcare approach to the management of chronic disease in Ethiopia: An example for other countries. Clin. Med..

[32] Li X., Krumholz H.M., Yip W., Cheng K.K., De Maeseneer J., Meng Q., Mossialos E., Li C., Lu J., Su M. (2020). Quality of primary health care in China: Challenges and recommendations. Lancet.

[33] Liu H., Fang C., Fan Y. (2020). Mapping the inequalities of medical resource provision in China. Reg. Stud. Reg. Sci..

[34] Zhang J., Han P., Sun Y., Zhao J., Yang L. (2021). Assessing Spatial Accessibility to Primary Health Care Services in Beijing, China. Int. J. Environ. Res. Public Health.

[35] Wan G., Wei X., Yin H., Qian Z., Wang T., Wang L. (2021). The trend in primary health care preference in China: A cohort study of 12,508 residents from 2012 to 2018. BMC Health Serv. Res..

[36] General Office of the State Council Key Tasks of Medical and Health System Reform in 2022. http://www.gov.cn/zhengce/content/2022-05/25/content_5692209.htm.

